# Association of Cell Death Markers With Tumor Immune Cell Infiltrates After Chemo-Radiation in Cervical Cancer

**DOI:** 10.3389/fonc.2022.892813

**Published:** 2022-07-12

**Authors:** Teodora Oltean, Lien Lippens, Kelly Lemeire, Caroline De Tender, Marnik Vuylsteke, Hannelore Denys, Katrien Vandecasteele, Peter Vandenabeele, Sandy Adjemian

**Affiliations:** ^1^ Cell Death and Inflammation Unit, Vlaams Instituut voor Biotechnologie (VIB)-UGent Center for Inflammation Research (IRC), Ghent, Belgium; ^2^ Department of Biomedical Molecular Biology (DBMB), Ghent University, Ghent, Belgium; ^3^ Ghent University, Cancer Research Institute Ghent (CRIG), Ghent, Belgium; ^4^ Laboratory of Experimental Cancer Research, Department of Human Structure and Repair, Ghent University, Ghent, Belgium; ^5^ Medical Oncology, Department of Internal Medicine and Pediatrics, Ghent University Hospital, Ghent, Belgium; ^6^ Department of Applied Mathematics, Computer Science and Statistics, Ghent University, Ghent, Belgium; ^7^ Plant Sciences Unit, Flanders Research Institute for Agriculture, Fisheries and Food (ILVO), Merelbeke, Belgium; ^8^ Gnomixx, Melle, Belgium; ^9^ Department of Radiation Oncology and Experimental Cancer Research, Ghent University, Ghent, Belgium; ^10^ Radiation Oncology, Ghent University Hospital, Ghent, Belgium; ^11^ Methusalem Program, Ghent University, Ghent, Belgium; ^12^ Vlaams Instituut voor Biotechnologie (VIB)-UGent Center for Inflammation Research (IRC) Vlaams Instituut voor Biotechnologie (VIB), Ghent, Belgium

**Keywords:** cervical cancer, biomarkers, immunogenic cell death, cell death, tumor infiltrating leucocytes

## Abstract

Irradiation induces distinct cellular responses such as apoptosis, necroptosis, iron-dependent cell death (a feature of ferroptosis), senescence, and mitotic catastrophe. Several of these outcomes are immunostimulatory and may represent a potential for immunogenic type of cell death (ICD) induced by radiotherapy triggering abscopal effects. The purpose of this study is to determine whether intra-tumoral ICD markers can serve as biomarkers for the prediction of patient’s outcomes defined as the metastasis status and survival over a 5-year period. Thirty-eight patients with locally advanced cervical cancer, treated with neoadjuvant chemoradiotherapy using cisplatin were included in this study. Pre-treatment tumor biopsy and post-treatment hysterectomy samples were stained for cell death markers and danger associated molecular patterns (DAMPs): cleaved caspase-3 (apoptosis), phosphorylated mixed lineage kinase domain like pseudokinase (pMLKL; necroptosis), glutathione peroxidase 4 (GPX4; ferroptosis) and 4-hydroxy-2-noneal (4-HNE; ferroptosis), high mobility group box 1 (HMGB1) and calreticulin. Although these markers could not predict the patient’s outcome in terms of relapse or survival, many significantly correlated with immune cell infiltration. For instance, inducing ferroptosis post-treatment seems to negatively impact immune cell recruitment. Measuring ICD markers could reflect the impact of treatment on the tumor microenvironment with regard to immune cell recruitment and infiltration.

**One Sentence Summary:**

Cell death readouts during neoadjuvant chemoradiation in cervical cancer

## Introduction

According to the World Health Organization (WHO), cervical cancer (CC) is the fourth most prevalent cancer in women worldwide. Standard of care for patients with locally advanced CC (LACC) usually consists in a chemoradiation regimen, with a dose of about 45-50 Gy using external beam radiotherapy on tumor, affected and elective lymph nodes, followed by a boost of sequential brachytherapy aiming for an on-tumor cumulative dose up to 90 Gy (1, 2). Ghent University Hospital investigated a different multimodality treatment schedule for LACC consisting in a higher dose per fraction on tumor, reaching a cumulative dose of 62 Gy with a simultaneous integrated boost (SIB) and surgery instead of brachytherapy (3). A pathologically complete response (pCR) is observed in 34% of patients 6–8 weeks post chemoradiotherapy (3, 4).

Since the discovery of X-rays by Röntgen in 1895, radiotherapy (XRT) was shown to improve clinical outcomes in many types of cancer (5). Today, XRT is used as main treatment for over 50% of cancer patients, or in combination with surgery, chemotherapy, and immunotherapy. Despite the potential side effects, XRT is still vividly used because of its efficacy. Moreover, in rare cases, XRT has been shown to initiate an “abscopal effect” in which non-irradiated metastatic tumors are eradicated after XRT is applied solely on the primary tumor, mostly when combined with immunotherapies (6). Although the full mechanisms are not yet elucidated, this effect is mediated by a systemic anti-tumor immune response (7, 8). We and others have previously shown that XRT can drive a variety of cellular outcomes such as senescence, mitotic catastrophe, apoptosis, necrosis, necroptosis, and lipid peroxidation (9, 10).

The cell death modality is decisive in the activation of the immune response. One of the strategies to increase the immune response against tumor cells is through the induction of immunogenic cell death (ICD) (11). ICD stimulates the immune response against tumor-specific antigens. Several anti-cancer treatments, such as anthracyclines and irradiation are able to stimulate the immune system by inducing an immunogenic apoptosis (12). Although apoptosis was initially identified as an homeostatic type of cell death, efficiently clearing cellular debris without eliciting an immune response (13–15), apoptosis can also be immunogenic when accompanied by the release of DAMPs (12, 16, 17). Necroptosis is a type of regulated necrosis, a cellular demise in response to extreme physiochemical stress and was shown to induce inflammation in several pathologies (18–20). This cell death pathway is mediated by receptor-interacting protein kinase-1 (RIPK1), RIPK3 and mixed lineage kinase domain like pseudokinase (MLKL). RIPK1 phosphorylates and activates RIPK3 which in turn phosphorylates the necroptosis executioner MLKL to destabilize the plasma membrane (21–24). Necroptosis leads to plasma membrane permeabilization and release of the intracellular content (25, 26). Upon irradiation, lipid peroxidation can also occur. Lipid peroxidation is known to drive a distinct type of programmed death, named ferroptosis (27). This type of cell death is iron-dependent and is driven by the loss of activity of the lipid repair enzyme glutathione peroxidase 4 (GPX4) (28). Therefore, high levels of GPX4 are expected to protect against lipid peroxidation, and less ferroptosis should occur. Levels of 4-hydroxynonenal (4-HNE), a product of lipid peroxidation, are expected to be high upon ferroptosis. Recently, it was suggested that ferroptosis could be partially immunogenic (7). Ferroptosis inducers can act as radiosensitizers (29). Moreover, radiation therapy was shown to induce lipid peroxidation (evidenced by 4-HNE staining) and detection of ferroptosis in cancer patients correlated with better response and survival (30).

DAMPs, including calreticulin, adenosine triphosphate (ATP) and high mobility group protein B1 (HMGB1), released from dying cells act as adjuvants and signal the state of danger to the organism (8, 15, 31). When exposed on the plasma membrane of cancerous cell undergoing ICD, calreticulin enhances phagocytosis by dendritic cells, a crucial step for tumor antigen presentation (32, 33). Extracellular HMGB1 (either released or secreted) initiates inflammation and activates the adaptive immune response (34, 35). *Hmgb1* gene expression is upregulated in most cervical cancers (36). It has been proposed that an increased expression level of HMGB1 is involved in the progression of squamous CC and could be used as a biomarker for patient prognosis (35).

A previous study (3) from our colleagues on the same set of patients already evaluated the scores of tumor infiltrating leucocytes (TILs): CD3 (T cells), CD4 (T helper), CD8 (present mostly on cytotoxic T cells, but also on a subset of macrophages and dendritic cells), FoxP3 (forkhead box P3; regulatory T cells), CD20 (B cells), CD68 (macrophage), and CD163 (present on type 2 macrophage), as well as the ionized calcium-binding adapter molecule 1 (Iba1; activated macrophages) and the two immuno-modulatory proteins, PD-L1 and interleukin-33 (IL-33). They found that patients with a high CD8 score in the biopsy had more pathological complete response and better cause-specific survival (CSS). In this retrospective study, we stained for cleaved caspase-3 as a marker of apoptosis, pMLKL as a marker for necroptosis. GPX4 staining was used to reflect antioxidant capacity protecting against ferroptosis as well as 4-HNE as a lipid peroxidation marker, crucial for driving ferroptosis. We also stained for two DAMPs: calreticulin and HMGB1, which are crucial markers of ICD. HMGB1 is also released following accidental necrosis, a non-regulated type of cell death likely to occur in human tumors (37). Due to the absence of specific markers of senescence and mitotic catastrophe, we did not assess these cellular outcomes. We aimed to assess whether the above-mentioned cell death markers and DAMPs correlated with immune cell infiltration, and to identify which type of TILs were associated to a specific cell death and DAMP marker. We also questioned whether our markers could predict the prognosis of patients in terms of relapse and 5-years survival post-treatment. We examined the expression of the markers in the biopsy tissues (pre-treatment), in the hysterectomy tissues (post-treatment) and the treatment-induced levels were determined with a subtraction between the hysterectomy levels and the levels in the biopsy. An additional correlation study between ICD markers and TILs was performed for the patients showing an incomplete pathological response.

## Material and Methods

### Patient Selection

Patient selection was previously described (3). All patients with LACC were included retrospectively (FIGO stage IB1-IVA and IVB amenable to curative therapy). These patients were diagnosed between 2006 and 2017 and had available for analysis biopsy and hysterectomy samples. All patients underwent radiotherapy with a simultaneously integrated boost on the tumor and affected lymph nodes if present. Dose and delivery of chemoradiotherapy were previously reported (38). More details on the addition of post-chemoradiotherapy surgery can be found in our previous report (39). This study was approved by the ethics committee of Ghent University Hospital (B670201732304) and the requirement to obtain informed consent was waived because of its retrospective nature. Treatment response was categorized into two categories: pathological complete response (pCR; no residual viable cancer cells, as assessed by the pathologist with a hematoxylin/eosin staining) and partial response (residual clusters of cancer cells, as assessed by the pathologist with a hematoxylin/eosin staining).

### Tumor Tissue Retrieval

Pre-treatment biopsies and post-treatment hysterectomies of 38 patients were obtained from the department of pathology of Ghent University Hospital (Ghent, Belgium). Each block contained formalin-fixed, paraffin-embedded tissue.

### Immunohistochemistry

Biopsy and hysterectomy tissue sections were deparaffinized and rehydrated according to routine procedures. Antigen retrieval was performed in a 2100 Retriever pressure cooker (PickCell Laboratories, Amsterdam, The Netherlands). A citrate buffer, pH6 (DAKO, S2031) was used for the following antibodies: calreticulin (Abcam, ab2907), HMGB1 (Abcam, ab18256), pMLKL (Abcam, ab196436), GPX4 (Abcam, ab125066) and cleaved caspase-3 (Abcam, ab2302). An EDTA buffer, pH 9 (Vector H-3301) was used for 4-HNE (Abcam, ab46545). Slides were washed 3 times for 5 min with PBS. Peroxidase was blocked with 3% H_2_O_2_ in methanol for 10 minutes. Following 3 additional washes with PBS, tissues were incubated with a blocking buffer (PBS containing 5% goat serum and 1% BSA) for 30 min. The blocking buffer was removed and sections were incubated overnight at 4°C with primary antibodies: calreticulin 1:1000, HMGB1 1:5000, pMLKL 1:1000, GPX4 1:1000, 4-HNE 1:1500 and cleaved caspase-3 1:100 in PBS containing 1% BSA. Slides were incubated with appropriate secondary antibodies (Dako,Glostrup, Denmark) and specific signals were enhanced by use of the ABC-kit (Vector Laboratories, Burlingame, USA). DAB (3, 3’-diaminobenzidine)(Vector) was used to detect the signal. The sections were counterstained with Hematoxylin, rinsed and dehydrated. Slides were mounted with a Xylene-based mounting medium. Images were acquired on a Zeiss Axio Scan.Z1 and ZEN 2 software (Zeiss). Images from the whole tissues were quantified with QuPath software (version 0.1.2) with a script enabling the identification of positively stained cells within the tissue. A percentage of positive cells per tissue was obtained with this quantification. The quantitative data of 38 patients were analyzed (1 patient diagnosed with an immunologic disorder was excluded) for correlations with tumor-infiltrating immune cells (TILs) and for changes between pre- and post-treatment samples. Staining of the same patient material (consecutive slicing) for TILs has been performed simultaneously in another project (3).

### Metastasis Status and Survival

Metastasis status was assessed at diagnosis and was followed *via* positron emission tomography with 2-deoxy-2-[fluorine-18] fluoro-D-glucose integrated with computed tomography (18-FDG PET/CT). Cause-specific survival (CSS) was used to analyze the prognostic value of the different markers therefore excluding four patients from the analysis who died due to intercurrent diseases. CSS was defined as the time between the end of therapy (i.e., surgery) and either the date of death from CC or the date of last follow-up and was analyzed over a 5-year period.

### Statistical Analysis

Two-sided test of correlation was done on the five markers quantified in this study (cleaved caspase-3, pMLKL, GPX4, 4-HNE, calreticulin, and HMGB1) and the TILs scores previously determined (3). We accounted for multiple testing by selecting a threshold p=0.05 corresponding to 0.17 FDR. P-values and correlations coefficients are shown on each graphic. This analysis was done in Genstat 64-bit Release 20.1.

Association analysis between the markers, the patients’ age, the lymph node status and FIGO stage (International Federation of Obstetrics and Gynecology), and the patients’ outcomes (relapse and CSS) was done in R, making use of a generalized linear model of the binomial family. For CSS, a generalized mixed effect model was used. The following mean models were used:


Relapse: logit(μ)~pMLKL+Calreticulin+GPX4+cleaved caspase 3+HMGB1+4HNE+Age+FIGO+N−status

CSS:logit(μ)~pMLKL+Calreticulin+GPX4+cleaved caspase 3+HMGB1+4HNE+Age+FIGO+(1/Months contact patient)

With µ the modeled mean. Both models contain all the main terms (fixed effects). In addition, the model on CSS corrected for the number of months between the last biopsy and last contact with the patient or death. Associations were corrected for multiple testing by controlling the false discovery rate at 5% making use of the Benjamini-Hochberg principle.

## Results

### Patients Baseline Characteristics

Baseline characteristics from these 38 patients were described previously (3). The mean age at diagnosis was 57 years. Thirty-four patients (89.5%) presented a squamous cell carcinoma, while 4 patients (10.5%) presented with an adenocarcinoma. Twenty-four patients (63.1%) presented a FIGO (2009) stage IIB. Half of the patients (50.0%) presented local lymph node metastasis at diagnosis, and 4 patients (10.5%) presented distant lymph node metastasis at diagnosis. Thirteen patients (34.2%) had a pathological complete response, and 25 patients (65.8%) had an incomplete response to the treatment. Nine patients died during the 5-year follow-up. Of these patients, 5 died due to cancer progression (2 patients died of a new, pathologically confirmed, non-gynecological cancer; 1 patient died of cardiac disease and 1 patient died without known cause but with no evidence of disease at the last follow-up moment).

### Levels of Cell Death Markers and DAMPs

**Figure 1:**
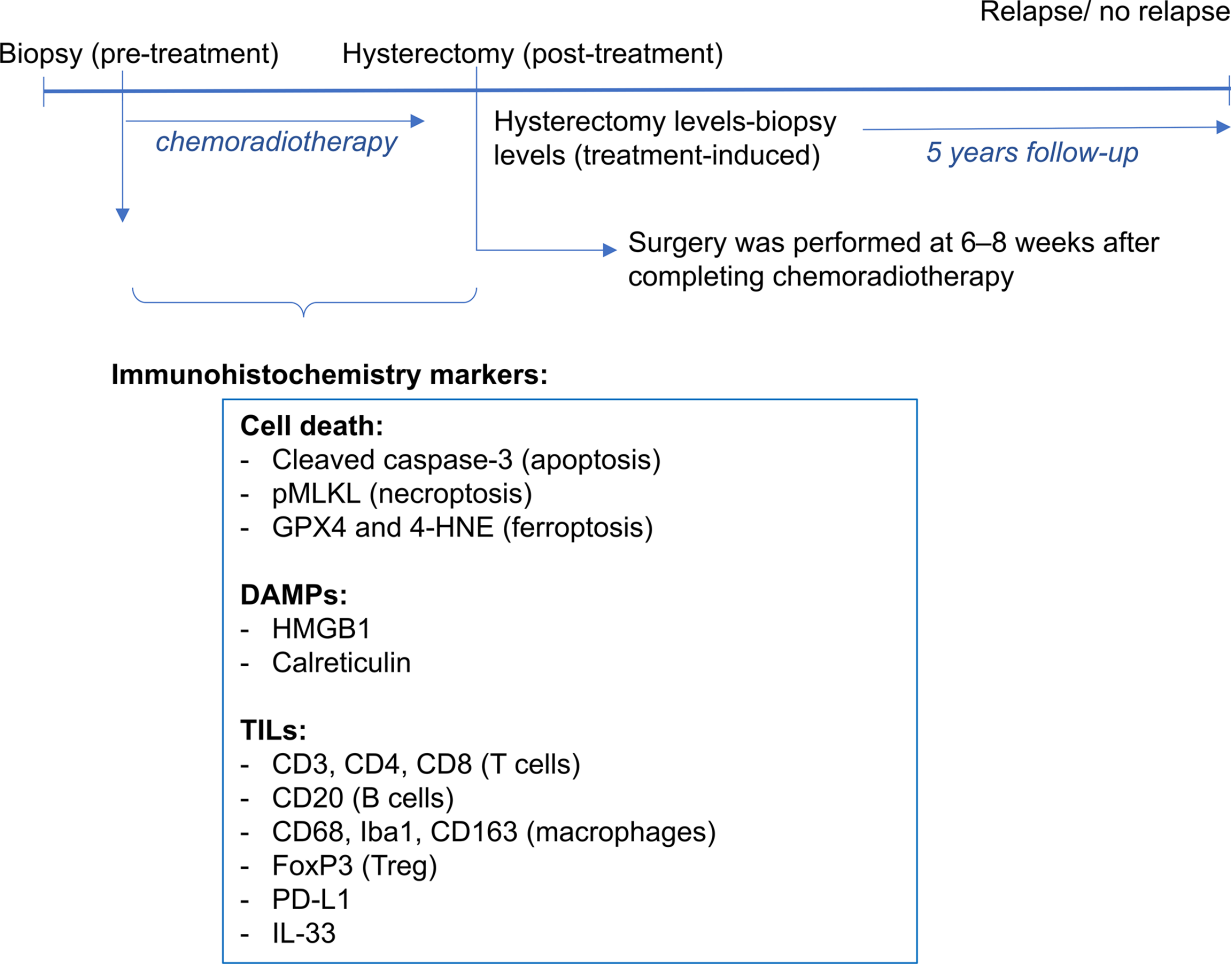
A scheme of the protocol to collect and stain tissue samples is shown. For each patient, a tissue block of the pre-treatment biopsy and post-treatment hysterectomy samples were obtained. Immunohistrochemistry markers were used for cell death and DAMPs (cleaved caspase-3 and pMLKL, GPX4 and 4-HNE, HMGB1 and calreticulin). A previous study assessed the tumor infiltrating leucocytes (CD3, CD4, CD8, CD20, CD68, Iba1, CD163, FoxP3, PD-L1 and IL-33). Patients were followed-up up to 5 years post-treatment, period in which survival and relapse were assessed.

**Figure 2:**
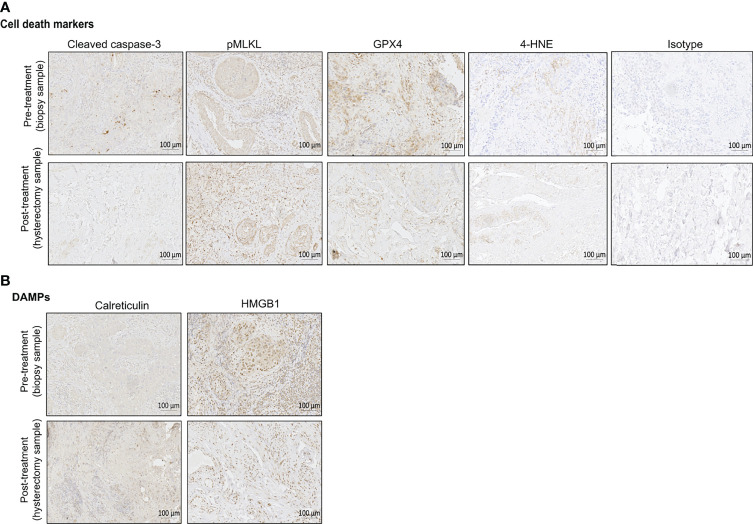
Representative immunohistochemical (IHC) staining of pre-treatment biopsy and post-treatment hysterectomy samples for the different cell death markers **(A)** and DAMPs **(B)**. Bright field images were taken with ZEISS Axio Scan.Z1 at a magnification of 20x. Scales bars are 100 µm as indicated on the images. One example of isotype control is also shown in this figure. For the pre-treatment biopsy, the percentages of positive cells per tissue slice are the following: cleaved caspase-3 (6,77%), pMLKL (53,12%), GPX4 (53,9%), 4-HNE (0,73%), calreticulin (7,24%), HMGB1 (66,37%). For the post-treatment hysterectomy, the percentages of positive cells per tissue slice are the following: cleaved caspase-3 (1,66%), pMLKL (31,42%), GPX4 (15,5%), 4-HNE (0,86%), calreticulin (52,10%), HMGB1 (34,53%).

A scheme of the protocol used is shown in **Figure 1**. Representative pictures of the immunohistochemistry slides are shown in **Figure 2**. In total, 25 patients (65.8%) had decreased pMLKL levels after chemoradiation treatment (**Table 1**). Most patients (68.4%) presented decreased levels of cleaved caspase-3, HMGB1 and GPX4 in their hysterectomy samples, as compared with their biopsies. Twenty-two patients (57.9%) had an increase in calreticulin post-treatment, and 27 patients (71.1%) had increased 4-HNE. These results suggest that necroptosis, apoptosis and accidental necrosis did not seem to be induced by chemoradiation in most patients. Ferroptosis, on the other hand might be induced by the treatment, as reduced levels of GPX4 might confer sensitivity to ferroptosis and increased 4-HNE levels might reflect augmented lipid peroxidation. Some patients seem to present more than one cell death modality induced upon treatment, as evidenced by the increase of more than one cell death marker in the hysterectomy samples as compared with the biopsy samples (data not shown).

**Table 1:** Levels of cell death/ICD markers in hysterectomy samples compared with biopsy samples.

Cell death/ICD marker	Increase n/N (%)	Decrease n/N (%)
**Calreticulin**	22/38 (57.8%)	16/38 (42.1%)
**HMGB1**	12/38 (31.6%)	26/38 (68.4%)
**Cleaved caspase-3**	12/38 (31.6%)	26/38 (68.4%)
**pMLKL**	13/38 (34.2%)	25/38 (65.8%)
**GPX4**	12/38 (31.6%)	26/38 (68.4%)
**4-HNE**	27/38 (71.1%)	11/38 (27.8%)

The number and percentage of patients showing an increase/decrease in the cell death/DAMP markers after the treatment (hysterectomy sample levels compared to initial biopsy sample levels) are shown for calreticulin, HMGB1, cleaved caspase-3, pMLKL, GPX4, and 4-HNE. n = number of patients with an increase or decrease of cell death/ICD marker in hysterectomy; N = total number of patients; pMLKL = phosphorylated mixed lineage kinase domain like pseudokinase; 4-HNE = 4-hydroxynonenal.

### Significant Correlations in Pre-Treatment Biopsy Samples – All Patients

**Figure 3:**
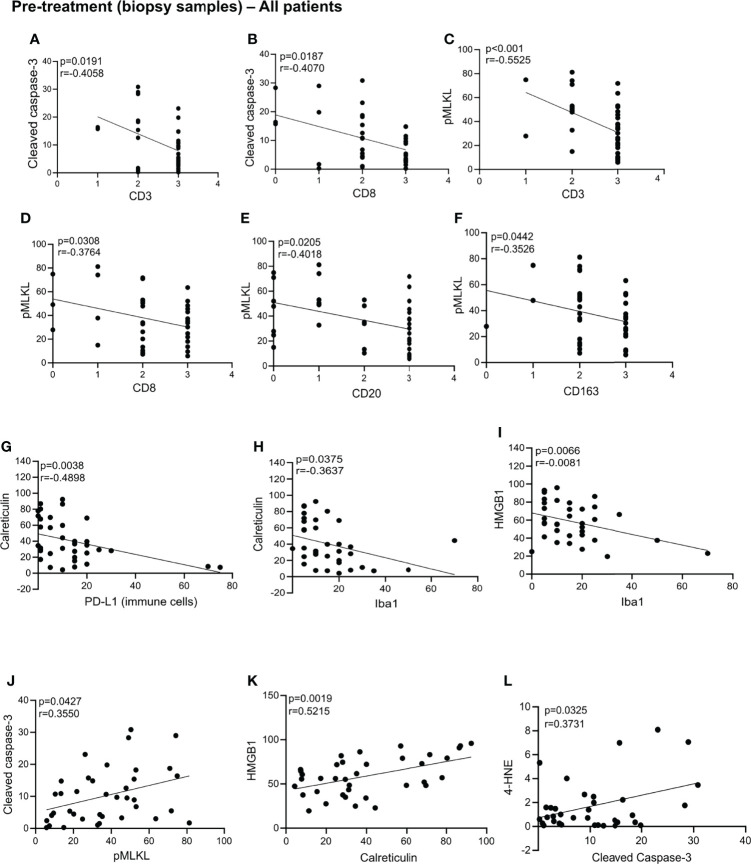
Statistically significant correlation in the biopsy samples. Statistically significant correlations were found between cleaved caspase-3 and CD3 **(A)**, CD8 **(B)**, CD3 **(C)**, CD8 **(D)**, CD20 **(E)**, CD163 **(F)**; calreticulin and PD-L1 **(G)** and Iba1 **(H)**; HMGB1 and Iba1 **(I)**; cleaved caspase-3 and pMLK **(J)**; HMGB1 and calreticulin **(K)**; 4-HNE and cleaved caspase-3 **(L)**. All p-values and correlation coefficients were evaluated with two-sided test of correlation (Genstat 64-bit Release 20.1) are indicated on the graphics which were generated with GraphPad (version 8).

We assessed whether cell death markers and DAMPs correlated with immune cell infiltration, relapse and survival in the biopsy samples. An overview of the statically significant correlations can be found in the **Supplemental Table 1**. In these pre-treatment biopsies, we found that patients levels of cleaved caspase-3 negatively correlated with levels of CD3, CD8 (**Figures 3A,B**). We also found that in the pre-treatment samples, levels of pMLKL negatively correlated with levels of CD3, CD8, CD20, CD163 (**Figures 3C–F**). These results suggest that apoptosis does not seem to be associated with the recruitment of CD8^+^ immune cells, while necroptosis was negatively associated with recruitment of CD8^+^ immune cells (T cells, macrophages or DCs), B cells and CD163^+^ cells (possibly M2 macrophages). Patients with high levels of calreticulin have lower PD-L1 expression on immune cells (**Figure 3G**). Patients with high levels of calreticulin and HMGB1 had lower levels of Iba1 (**Figures 3H,I**). Pre-treatment samples with high levels of cleaved caspase-3 also showed high levels of pMLKL and 4-HNE (**Figures 3J,L**), suggesting that apoptosis might co-exist with necroptosis and ferroptosis in tumors prior to treatment. High levels of HMGB1 correlated with high levels of calreticulin (**Figure 3K**). No association between the cell death markers, DAMPs, FIGO stage, age or lymph node status with relapse and CSS were found in the biopsies (**Supplemental Table 2**).

### Significant Correlations in Post-Treatment Hysterectomy Samples – All Patients

**Figure 4:**
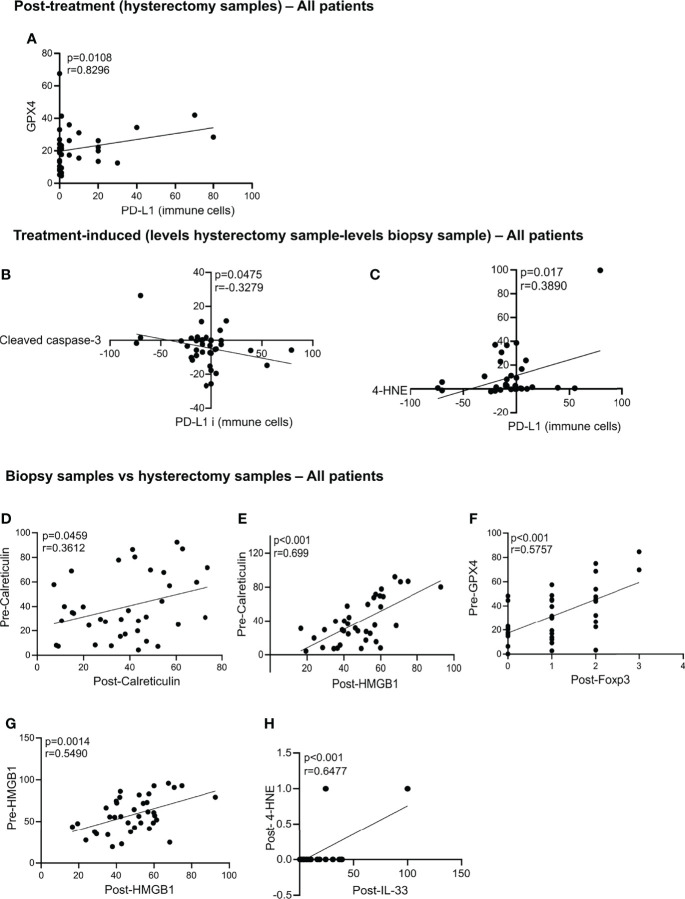
Statistically significant correlation in the hysterectomy samples, treatment-induced and biopsy samples vs hysterectomy samples. Statistically significant correlations were found post-treatment between GPX4 and PD-L1 **(A)**. Correlation in the treatment-induced analysis between PD-L1 and cleaved caspase-3 **(B)** and 4-HNE and PD-L1 **(C)**. Correlations between the biopsy samples vs hysterectomy samples in all patients are significant for pre-calreticulin and post-calreticulin **(D)**; pre-calreticulin and post-HMGB1 **(E)**; pre-GPX4 and post-FoxP3 **(F)**; pre-HMGB1 and post-HMGB1 **(G)** and post-4-HNE and post-IL-33 **(H)**. All p-values and correlation coefficients were evaluated with two-sided test of correlation (Genstat 64-bit Release 20.1) are indicated on the graphics which were generated with GraphPad (version 8).

In the post-treatment samples, we observed that increased levels of GPX4 are correlated with more PD-L1 expression on immune cells in the post-treatment samples (**Figure 4A**). This observation could suggest that an increase in the antioxidant capacity of GPX4 protecting against ferroptosis is associated with increased PD-L1 on immune cells, which might suggest increased immune resistance. No significant associations were found between the cell death markers, DAMPS, Age, FIGO stage and node status with relapse and CSS in the hysterectomy samples (**Supplemental Table 2**). A trend towards an association between pMLKL and survival was found, although not significant after multiple testing, indicating that a higher sample size to increase the power might be sufficient to prove this association.

### Treatment-Induced Significant Correlations – All Patients

To identify the markers that were induced by the treatment, we subtracted the levels in the hysterectomy sample from the biopsy sample levels. We found that treatment-induced increased cleaved caspase-3 is associated with a decrease in PD-L1 expression on immune cells (**Figure 4B**). Contrarily, an increase in the 4-HNE by the treatment is associated with an increase of PL-L1 expression on immune cells (**Figure 4C**). No correlations between the cell death markers, DAMPs with No correlation between the cell death markers with relapse, age, FIGO stage, lymph node status and CSS were found (**Supplemental Table 2**).

### Significant Correlations Biopsy Samples vs. Hysterectomy Samples – All Patients

Next, we assessed whether there was a correlation between the levels found in the pre-treatment biopsy samples and the post-treatment hysterectomy samples. We found that patients with high levels of calreticulin in the biopsy samples also had high levels of calreticulin and HMGB1 after treatment (**Figures 4D,E**). High levels of GPX4 in the biopsies correlated with high infiltration of Treg (high levels of FoxP3) in the post-treatment hysterectomy samples (**Figure 4F**). Patients with high HMGB1 levels in their biopsy had higher HMGB1 levels after chemoradiation (**Figure 4G**). Post-treatment levels of 4-HNE were positively correlated with the levels of IL-33 (**Figure 4H**).

### Post-Treatment and Treatment-Induced Significant Correlations – Patients With Incomplete Response

**Figure 5:**
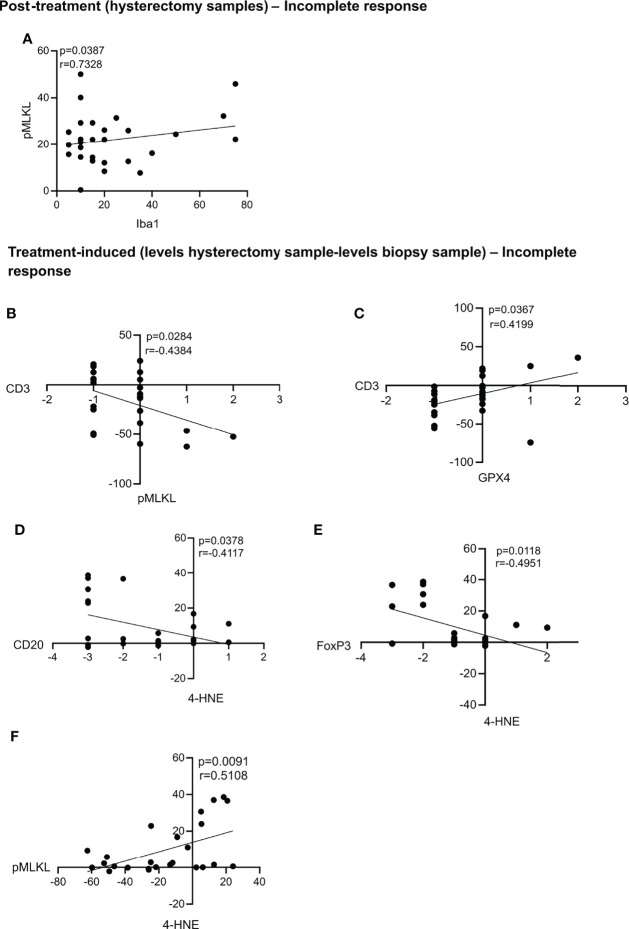
Statistically significant correlations in the post-treatment samples and treatment-induced analysis for patients with an incomplete response. Statistically significant correlations were found between pMLKL and Iba1 in the post-treatment samples **(A)**. Correlations between the treatment-induced levels of pMLKL and CD3 **(B)**, GPX4 and CD3 **(C)**; 4-HNE and CD20 **(D)** and FoxP3 **(E)** and pMLKL and 4-HNE **(F)** were also statistically significant. All p-values and correlation coefficients were evaluated with two-sided test of correlation (Genstat 64-bit Release 20.1) are indicated on the graphics which were generated with GraphPad (version 8).

In patients with an incomplete response, we found a positive correlation between the levels of pMLKL and Iba1 in the post-treatment, hysterectomy samples (**Figure 5A**). Also, in the treatment-induced analysis, we found that most patients with an incomplete response had a decrease of pMLKL in the hysterectomy sample compared to the biopsy sample, and this correlated with an increase in CD3 levels induced by chemoradiation (**Figure 5B**). We also found that treatment-induced levels of GPX4 were associated with more CD3 cells in the patients with an incomplete response to the treatment (**Figure 5C**). Increased levels of 4-HNE, which could indicate lipid peroxidation induced by chemoradiation were correlated with decreased levels of CD20 and FoxP3 (**Figures 5D,E**). Increased levels of 4-HNE upon chemoradiation were also correlated with increased pMLKL levels, suggesting that chemoradiation could trigger ferroptosis and necroptosis in the same patients (**Figure 5F**).

### Cause-Specific Survival and Relapse

Although we did not find any association between the cell death markers, DAMPs and cause-specific survival and relapse after 5 years follow-up, we observed some trends (p< 0.1). In both biopsy and hysterectomy samples, we observed a trend towards higher levels of calreticulin and HMGB1 in patients who died or relapsed (patients with CSS = 1 and Relapse = 1) (**Supplementary Figure 1**).

## Discussion

Compelling evidence suggests that the clinical success of some cancer therapies is not solely due to tumor cell toxicity but relies on the activation of the immune system for a long-term effect (15, 32). ICD can prime the immune system to fight against cancer *via* two mechanisms: antigenicity and adjuvanticity. Antigenicity depends on the high level of mutations in cancers to produce the so-called tumor-associated antigens (TAAs) which are recognized by the immune system as non-self. Adjuvanticity is modulated by DAMPs such as calreticulin and HMGB1 which act as adjuvants to boost the anti-cancer immune response (40). Several *in vitro* and *in vivo* animal studies have shown the importance of ICD for a long-term success of anti-cancer therapies (15). Other studies also assessed the presence of ICD markers in the tumors of cancer patients and have reported conflicting results (41). A decreased expression of HMGB1 and increase expression of CRT was reported to be beneficial for the survival of patients with gliobastoma multiforme (42). On the other hand, HMGB1 levels were found to increase following chemoradiation only in patients with locally advanced head and neck squamous cell carcinoma who did not relapse, as opposed to the patients who relapsed (43). In patients with esophageal squamous cell carcinoma, HMGB1 was found to positively correlate with patient survival and was found upregulated in the tumor following preoperative chemoradiotherapy (29).

In this study, we wanted to investigate whether key markers of cell death and ICD could serve as biomarkers to predict the therapeutic response of CC patients to chemoradiotherapy and whether a cell death modality was associated with the recruitment of a particular type of immune cells. We found that none of the ICD/DAMP markers could serve as prognostic biomarkers for the patient’s outcomes neither in the pre or post-treatment samples in this cohort of patients. However, several interesting correlations were found between the different ICD and DAMP markers and the immune infiltrates.

First, we found that necroptosis, apoptosis and accidental necrosis did not seem to be induced by chemoradiation in most patients. The hysterectomies were performed six to eight weeks after the end of the treatment and this timing could be too late to observe tumor cell death. Following the chemoradiation treatment we found low levels of GPX4, which might confer sensitivity to ferroptosis and increased 4-HNE levels, which might reflect increase lipid peroxidation. Radiation therapy leads to ROS generation and upregulated antioxidant systems including the transcription factor NF-E2-related factor 2 (Nrf2), a key regulator of the antioxidant system and leading to radioresistance (44). The oxidative stress results in oxidation of polyunsaturated fatty acids (PUFAs) through free radical chain reactions forming eventually lipid peroxidation (LPO) products, such as 4-HNE (45). Nrf2-induced genes counteract ferroptosis sensitivity (46). This intricate relationship between radiation-mediated redox modulation and ferroptosis sensitivity makes it impossible to attribute the modulation of 4-HNE and GPX4 to ferroptosis or redox modulation by the treatment. Some patients seem to present more than one cell death modality induced upon treatment. These results are in line with several *in vitro* and in-patient studies showing that radiotherapy, with or without chemotherapy led to several types of cell death modalities (47). We have also previously shown that lipid peroxidation and iron-dependent cell death were induced in a panel of cancer cell lines following radiotherapy (9).

Additionally, we found that some cell death markers and DAMPs were significantly correlated with distinct TILs in the biopsy as well as the hysterectomy samples. In the pre-treatment samples, we found that high levels of immune cells in the tumor site were not associated with apoptosis nor necroptosis. Thus, in the biopsy samples, before treatment, it seems that a strong immune cell infiltration is installed in the absence of cell death such as apoptosis and necroptosis. One hypothesis could be that this is the result of an efficient killing, phagocytosis of dying tumor cells, or as mentioned above, tumor cell death is no longer detected. The presence of DAMPs was correlated with a low PD-L1 expression on immune cells and lower levels of activated macrophages. Since PD-L1 downregulates the immune response in the context of cancers, a low expression might be beneficial for the patient. Furthermore, the low level of PD-L1 observed on immune cells could also be due to a lower amount of immune cells in the post-treatment samples.

When we assessed the changes in levels found in hysterectomy samples as compared with the biopsy samples, we found that increased post-treatment levels of cleaved caspase-3 were associated with a decrease in the PD-L1 expression on immune cells post-treatment. This result Underlines a positive role of treatment-induced apoptosis in these patients since the decrease in PD-L1 levels might lead to a better anti-tumoral immune response. On the other hand, an increase in lipid peroxidation was associated with an increase in PD-L1, suggesting that ferroptosis would not have a positive impact in the patients.

In the patients with an incomplete response to the treatment, increased levels of 4-HNE, which could indicate lipid peroxidation induced by chemoradiation correlated with decreased levels of TILs (CD20 and FoxP3). Also, treatment-induced increased in 4-HNE correlated with an increase in PD-L1 on immune cells. We recently reported that ferroptosis impedes antigen presentation. After this sentence, we could add “we recently reported that cancer cells dying from ferroptosis impede dendritic cell-mediated anti-tumor immunity”. Citation: Wiernicki B, Maschalidi S, Pinney J, Adjemian S, Vanden Berghe T, Ravichandran K, Vandenabeele P. Cancer cells dying from ferroptosis impede dendritic cell-mediated anti-tumor immunity. Nature Communications. In press. In line with this, high GPX4 expression was also shown to be correlated with better survival and induction of an immune response (48). Moreover, according to the human protein atlas, high GPX4 expression in cervical cancer is associated with a favorable clinical outcome. Indeed, GPX4 is an important detoxifying enzyme that reduces lipid peroxides, thus preventing the cells from undergoing ferroptosis. GPX4 increase could be beneficial to reduce cancer-associated oxidative stress. Increased levels of 4-HNE upon chemoradiation also correlated with increased pMLKL, suggesting that chemoradiation could trigger ferroptosis and necroptosis in the same patients. Patients presenting high levels of DAMPs prior treatment also had high levels of DAMPs after treatment, suggesting that the chemoradiation regimen was not able to modulate DAMPs release in these patients, and rather had no impact on this event. The combination of radiotherapy and cisplatin has been tested previously in the context of immunogenic cell death, and several studies outline an immunogenic effect of this combination (29, 49–52). In these studies, the combination of radiotherapy and cisplatin were shown to be immunogenic and improve the efficacy of immunotherapy. Currently, several clinical trials for cervical cancer are testing the combination of radiotherapy or chemoradiation with immunotherapies using similar radiotherapy regimen as the one used in this study (53).

To date, up to 30% of patients of LACC patients treated with CRT will suffer a recurrence with a subsequent short life expectancy and only few treatment options. Currently, all LACC patients are treated equally, both in the primary and recurrent setting. Biomarkers will allow us to evolve towards a personalized and patient-individualized setting with potentially radiation dose-(de)escalation in responders/non-responders and addition of chemotherapy, immunotherapy (anti-PD(L)1) and targeted therapy in those patients predicted to recur. Identification of biomarker data that can help to enrich the population that is most likely to benefit, would be highly beneficial and avoid treatment-related toxicity (also financial) in those patients that will not benefit. Gaining insight in the mechanisms of treatment response and correlation with tumor immune status may enable research towards new synergistic treatment combinations for the optimal priming of the immune system or even new treatment pathways.

Altogether, our results suggest that features of ferroptotic cell death seem to be induced following chemoradiation and that ferroptosis (decreased GPX4, increased HNE-4) post-treatment seems to negatively impact immune cell recruitment. Although not induced in the majority of patients, apoptosis and necroptosis also occurred upon chemoradiation, and several patients presented more than one cell death modality.

Our study presents several limitations. The sample size is small, with 38 patients included. A sample size calculation using Gpower (z-test; 2-sided; logistic regression with continuous predictor) showed that in function of the significance level, 42 up to 99 patients are required to detect an association between CSS and pMLKL with a power of 0.800. Next to age and FIGO stage, other factors might also influence the data (such as BMI, smoking habits, etc.), and it would have been interesting to analyze their correlation with survival, relapse and biomarkers. The markers used in this study also have cell-death independent functions, and thus might not reflect accurately the levels of intra-tumor death. Despite these shortcomings, we believe that this study is a first attempt to show the association between cell cell death markers and immune cells in cervical cancer. Our findings should be confirmed by a study with a larger experimental setup.

As the treatment of cancer becomes more and more personalized, monitoring the interplay between tumor cells and the immune microenvironment is becoming increasingly important and provide clues about how each tumor respond to the treatment. Systematic studies exploring treatment regimen where tumor tissues are retrieved prior and after treatment could contribute to this knowledge and might help in selecting more appropriate treatments.

## Data Availability Statement

The original contributions presented in the study are included in the article/**Supplementary Material**. Further inquiries can be directed to the corresponding author.

## Ethics Statement

The studies involving human participants were reviewed and approved by Ghent University Hospital (B670201732304). The patients/participants provided their written informed consent to participate in this study.

## Author Contributions

SA, PV, and KV designed and supervised the study. KV supervised sample collection and clinical annotation. TO, KL, and SA performed immunohistochemistry staining. TO and SA made the figures. MV and CT performed data analysis. TO, SA, and PV wrote the manuscript. HD, SA, and LL contributed to critical data interpretation. All of the authors have read and provided comments on the manuscript. All authors contributed to the article and approved the submitted version.

## Funding

This work was supported by a young-investigators-proof-of-concept grant from the CRIG obtained by KV. TO held a doctoral fellowship from FWO (Flanders Research Organization) (1S72616N). KV held a mandate for clinical and translational research funded by the Foundation against Cancer (Stichting tegen Kanker). SA held a post-doctoral fellowship from FWO. CDT holds an FWO grant (12S9418N). LL is funded by an Emmanuel van der Schueren scholarship of KOTK. PV is senior full professor at Ghent University and senior PI at the VIB-UGent Center for Inflammation Research (IRC). Research in the Vandenabeele group is supported by EOS MODEL-IDI (30826052), EOS- CD-INFLADIS (40007512), FWO research grants (G.0E04.16N, G.0C76.18N, G.0B71.18N, G.0B96.20N, G.0A93.22N), Methusalem (BOF16/MET_V/007), iBOF20/IBF/039 ATLANTIS, Foundation against Cancer (FAF-F/2016/865, F/2020/1505), CRIG and GIGG consortia, and VIB.

## Conflict of Interest

Author MV was employed by Gnomixx.

The remaining authors declare that the research was conducted in the absence of any commercial or financial relationships that could be construed as a potential conflict of interest.

## Publisher’s Note

All claims expressed in this article are solely those of the authors and do not necessarily represent those of their affiliated organizations, or those of the publisher, the editors and the reviewers. Any product that may be evaluated in this article, or claim that may be made by its manufacturer, is not guaranteed or endorsed by the publisher.

## Supplementary Material

Supplementary Table 1 An overview of the statically significant correlations between the cell death markers/DAMPs and the TILs. Significant correlations between the levels of the different cell death/DAMPs measured in this study and the TILs (3) are shown in all patients in the biopsy samples, the treatment-induced levels (hysterectomy sample levels - biopsy sample levels), as well as the pre- vs. post-treatment (biopsy sample levels vs. hysterectomy sample levels). Patients with an incomplete response showing significant correlations between the cell death markers/DAMPs and the TILs are shown in hysterectomy sample and the treatment-induced levels (hysterectomy sample levels - biopsy sample levels). P-values and correlation coefficients are shown for each association. DAMPs = damage-associated molecular patterns; TILs = Tumor-infiltrating Leukocytes; pMLKL = phosphorylated mixed lineage kinase domain like pseudokinase; HMGB1 = High Mobiliy Group Box 1; 4-HNE = 4-hydroxynonenal; PD-L1 immune = programmed death-ligand 1 expressed on immune cells; GPX4 = glutathione peroxidase 4; Iba1 = ionized calcium binding adaptor molecule 1.

Supplementary Table 2 Association between cell death markers, DAMPs, Age, FIGO, Lymph-node status with Relapse or CSS. Association between the cell death markets, DAMPs, Age, FIGO and lymph-node status with Relapse or CSS are shown in the biopsy samples (pre-treatment), the hysterectomies (post-treatment) and the treatment-induced levels (hysterectomy sample levels - biopsy sample levels). P-values and p-values FDR-corrected are shown for each association. DAMPs, damage-associated molecular patterns; pMLKL, phosphorylated mixed lineage kinase domain like pseudokinase; HMGB1, High Mobiliy Group Box 1; 4-HNE, 4-hydroxynonenal; GPX4, glutathione peroxidase 4; FIGO, (International Federation of Obstetrics and Gynecology).
