# Supplementary material for: Association of Cell Death Markers With Tumor Immune Cell Infiltrates After Chemo-Radiation in Cervical Cancer
Source: Front Oncol. 2022 Jul 12;12:892813. doi: 10.3389/fonc.2022.892813 (PMC9316180; doi:10.3389/fonc.2022.892813)
Supplement: Supplementary Table 1 — An overview of the statically significant correlations between the cell death markers/DAMPs and the TILs. Significant correlations between the levels of the different cell death/DAMPs measured in this study and the TILs (3) are shown in all patients in the biopsy samples, the treatment-induced levels (hysterectomy sample levels - biopsy sample levels), as well as the pre- vs. post-treatment (biopsy sample levels vs. hysterectomy sample levels). Patients with an incomplete response showing significant correlations between the cell death markers/DAMPs and the TILs are shown in hysterectomy sample and the treatment-induced levels (hysterectomy sample levels - biopsy sample levels). P-values and correlation coefficients are shown for each association. DAMPs = damage-associated molecular patterns; TILs = Tumor-infiltrating Leukocytes; pMLKL = phosphorylated mixed lineage kinase domain like pseudokinase; HMGB1 = High Mobiliy Group Box 1; 4-HNE = 4-hydroxynonenal; PD-L1 immune = programmed death-ligand 1 expressed on immune cells; GPX4 = glutathione peroxidase 4; Iba1 = ionized calcium binding adaptor molecule 1. [file DataSheet_1.pdf]

Supplemental Tables:

Overview of significant correlations

**Correlations cell death markers/DAMPs/TILs in biopsy samples – All patients**		**p-value**	**Correlation coefficient**
Cleaved caspase-3	CD3	0.0191	-0.4058
Cleaved caspase-3	CD8	0.0187	-0.4070
pMLKL	CD163	0.0442	-0.3526
pMLKL	CD20	0.0205	-0.4018
pMLKL	CD3	<0.001	-0.5525
pMLKL	CD8	0.0308	-0.3764
pMLKL	Cleaved caspase-3	0.0427	0.3550
Calreticulin	PD-L1 immune cells	0.0038	-0.4898
Calreticulin	Iba1	0.0375	-0.3637
HMGB1	Iba1	0.0066	-0.4637
HMGB1	Calreticulin	0.0019	0.5215
4-HNE	Cleaved caspase-3	0.0325	0.3731

**Correlations cell death markers/DAMPs/TILs in hysterectomy samples– All patients**		**p-value**	**Correlation coefficient**
GPX4	PD-L1 immune cells	0.0108	0.8296

**Correlations cell death markers/DAMPs/TILs Treatment-induced levels – All patients**		**p-value**	**Correlation coefficient**
Cleaved caspase-3	PD-L1 immune cells	0.0475	-0.3279
4-HNE	PD-L1 immune cells	0.0173	0.3891

**Correlations cell death markers/DAMPs/TILs in biopsy samples vs hysterectomy samples – All Patients**		**p-value**	**Correlation coefficient**
Pre-GPX4	Post-Foxp3	<0.001	0.5757
Pre-Calreticulin	Post-Calreticulin	0.0459	0.3612
Pre-Calreticulin	Post-HMGB1	<0.001	0.6966
Pre-HMGB1	Post-HMGB1	0.0014	0.5490

**Correlations cell death markers/DAMPs/TILs in hysterectomy samples – Incomplete response**		**p-value**	**Correlation coefficient**
pMLKL	Iba1	0.0387	0.7328

**Correlations cell death markers/DAMPs/TILs Treatment-induced levels – Incomplete response**		**p-value**	**Correlation coefficient**
GPX4	CD3	0.0367	0.4199
pMLKL	CD3	0.0284	-0.4384
4-HNE	CD20	0.0378	-0.4117
4-HNE	FoxP3	0.0118	-0.4951
4-HNE	pMLKL	0.0091	0.5108

Supplemental Table 1. An overview of the statically significant correlations between the cell death markers/DAMPs and the TILs. Significant correlations between the levels of the different cell

death/ DAMPs measured in this study and the TILs[3] are shown in all patients in the biopsy samples, the treatment-induced levels (hysterectomy sample levels - biopsy sample levels), as well as the pre- vs. post-treatment (biopsy sample levels vs. hysterectomy sample levels). Patients with an incomplete response showing significant correlations between the cell death markers/DAMPs and the TILs are shown in hysterectomy sample and the treatment-induced levels (hysterectomy sample levels - biopsy sample levels). P-values and correlation coefficients are shown for each association. DAMPs = damage-associated molecular patterns; TILs = Tumor-infiltrating Leukocytes; pMLKL = phosphorylated mixed lineage kinase domain like pseudokinase; HMGB1 = High Mobiliy Group Box 1; 4-HNE = 4-hydroxynonenal; PD-L1 immune = programmed death-ligand 1 expressed on immune cells; GPX4 = glutathione peroxidase 4; Iba1 = ionized calcium binding adaptor molecule 1.

**Association cell death markers/DAMPs/Age/FiGO/Lymph-node satus with Relapse or CSS in biopsy samples**		**p-value**	**p-value FDR corrected**
pMLKL	Relapse	0,97669	0,98
Calreticulin	Relapse	0,082	0,867
GPX4	Relapse	0,62476	0,948
Cleaved Caspase 3	Relapse	0,8396	0,968
HMGB1	Relapse	0,44347	0,879
4-HNE	Relapse	0,20865	0,867
Age	Relapse	0,40006	0,867
FIGO	Relapse	0,51117	0,928
Lymph-node status	Relapse	0,10469	0,868
pMLKL	CSS	0,973	0,98
Calreticulin	CSS	0,1467	0,867
GPX4	CSS	0,1909	0,867
Cleaved Caspase 3	CSS	0,7365	0,948
HMGB1	CSS	0,4559	0,879
4-HNE	CSS	0,9602	0,98
Age	CSS	0,8425	0,968
FIGO	CSS	0,2054	0,867
Lymph-node status	CSS	0,3838	0,867

**Association cell death markers/DAMPs/Age/FiGO/Lymph-node satus with Relapse or CSS in hysterectomy samples**		**p-value**	**p-value FDR corrected**
pMLKL	Relapse	0,7346	0,9482
Calreticulin	Relapse	0,421	0,874
GPX4	Relapse	0,3118	0,867
Cleaved Caspase 3	Relapse	0,3681	0,867
HMGB1	Relapse	0,3805	0,867
4-HNE	Relapse	0,9189	0,979
Age	Relapse	0,5734	0,938
FIGO	Relapse	0,1978	0,867
Lymph-node status	Relapse	0,3371	0,867
pMLKL	CSS	**0,02687***	0,867
Calreticulin	CSS	0,40155	0,867
GPX4	CSS	0,81961	0,968
Cleaved Caspase 3	CSS	0,67561	0,948
HMGB1	CSS	0,38296	0,866
4-HNE	CSS	0,70889	0,948
Age	CSS	0,6747	0,948
FIGO	CSS	0,22307	0,868
Lymph-node status	CSS	0,64231	0,948

**Association cell death markers/DAMPs/Age/FiGO/Lymph-node satus with Relapse or CSS – Treatment-induced levels**		**p-value**	**p-value FDR corrected**
pMLKL	Relapse	0,9797	0,98
Calreticulin	Relapse	0,1513	0,867
GPX4	Relapse	0,7756	0,952
Cleaved Caspase 3	Relapse	0,355	0,867
HMGB1	Relapse	0,2501	0,867
4-HNE	Relapse	0,9625	0,979
Age	Relapse	0,6591	0,948

FIGO	Relapse	0,1928	0,867
Lymph-node status	Relapse	0,1251	0,867
pMLKL	CSS	0,3469	0,867
Calreticulin	CSS	0,39	0,867
GPX4	CSS	0,5645	0,938
Cleaved Caspase 3	CSS	0,5154	0,928
HMGB1	CSS	0,768	0,952
4-HNE	CSS	0,7375	0,948
Age	CSS	0,878	0,98
FIGO	CSS	0,2253	0,867
Lymph-node status	CSS	0,5577	0,938

Supplemental Table 2. Association between cell death markers, DAMPs, Age, FIGO, Lymph-node status with Relapse or CSS. Association between the cell death markets, DAMPs, Age, FIGO and lymph-node status with Relapse or CSS are shown in the biopsy samples (pre-treatment), the hysterectomies (post-treatment) and the treatment-induced levels (hysterectomy sample levels - biopsy sample levels). P-values and p-values FDR-corrected are shown for each association. DAMPs = damage-associated molecular patterns; pMLKL = phosphorylated mixed lineage kinase domain like pseudokinase; HMGB1 = High Mobiliy Group Box 1; 4-HNE = 4-hydroxynonenal; GPX4 = glutathione peroxidase 4; FIGO = (International Federation of Obstetrics and Gynecology).

Supplemental Figure:

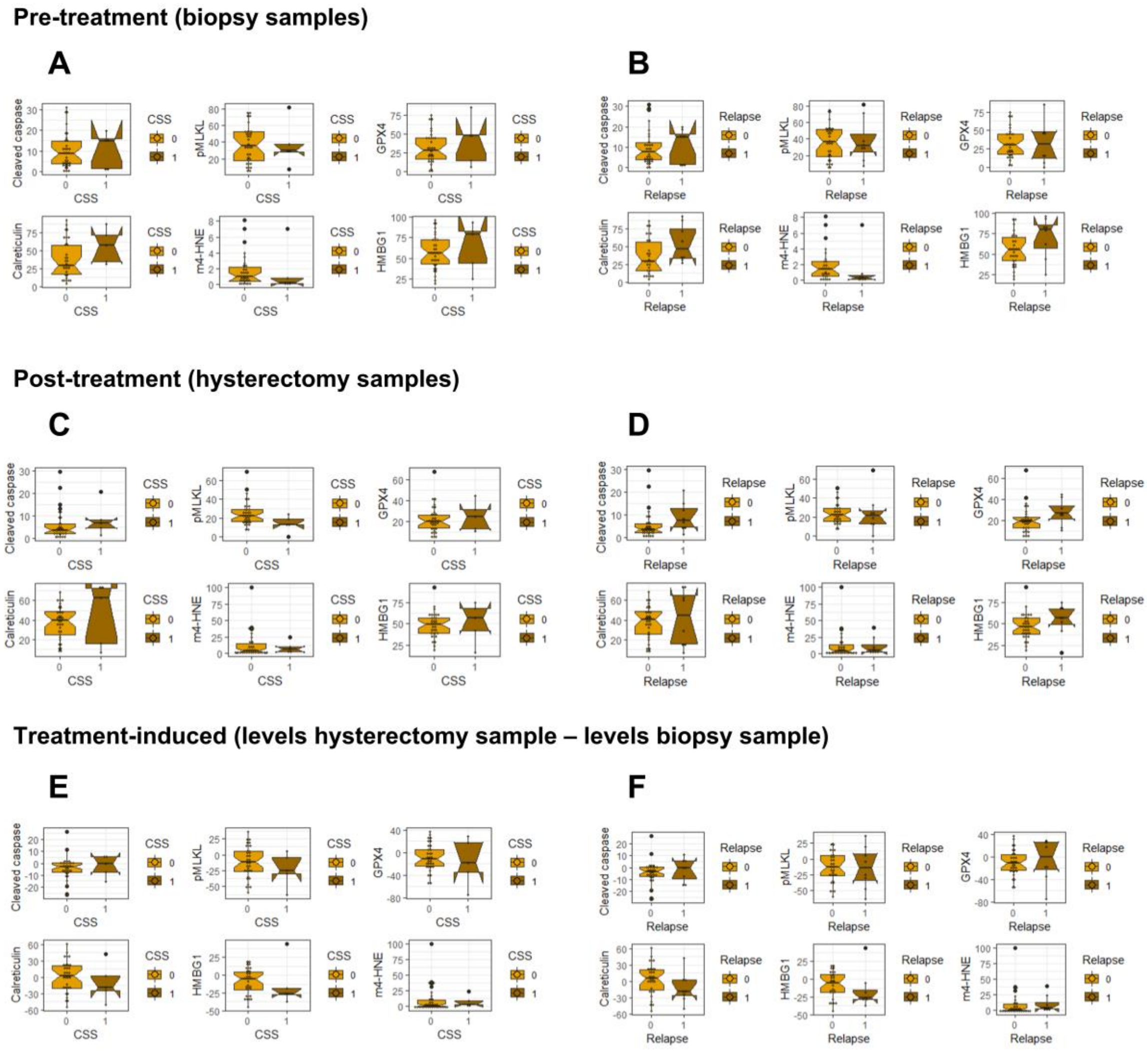

Supplemental figure 1. Notched boxplots representing ICD markers in relation to the outcome of the patients. All illustrative representations are shown pre-treatment (biopsy samples; panels A, B), post-treatment (hysterectomy samples; panels C, D), and treatment-induced (levels hysterectomy samples - levels biopsy samples; panels E, F). Cell death parameters (cleaved caspase-3, pMLKL, GPX4, Calreticulin, 4-HNE, HMGB1) are compared in function of the outcome of the patients: Cancer Specific Survival (CSS) (0, 1; panels A, C, E) and Relapse (0, 1; panels B, D, F). The x-axis represents the outcome of the patient with “0” in case of no CSS and no Relapse, and “1” in case of CSS and Relapse. The y-axis represents the quantitative data of the cell death parameters as indicated based on staining of samples from 38 patients pre-treatment (biopsy samples; panels A, B) and post-treatment (hysterectomy samples; panels C, D). The panels E, F illustrate the difference between post- and pre-treatment. The confidence interval for each box is shown on the median value, and indicated as a notch.

In some cases (Calreticulin pre- and post-treatment CSS and Relapse, HMGB1 pre- and post-treatment CSS and Relapse) the notches do not overlap, indicating a potential effect or trend between the groups. This could however not be verified as no statistical significance was noticed using GMM (binomial family) analysis.
